# Protein Design Meets Single-Molecule Detection: Towards Programmable Nanopore Sensors

**DOI:** 10.3390/ijms262110561

**Published:** 2025-10-30

**Authors:** Xintong Liu, Chunfu Xu

**Affiliations:** 1 Tsinghua Institute of Multidisciplinary Biomedical Research, Tsinghua University, Beijing 102206, China; liuxintong@nibs.ac.cn; 2 Peking University-Tsinghua University-National Institute of Biological Sciences Joint Graduate Program, Beijing 102206, China; 3 National Institute of Biological Sciences, Beijing 102206, China

**Keywords:** nanopore sequencing, *de novo* protein design, single-molecule detection

## Abstract

Nanopores have emerged as powerful tools for single-molecule detection, enabling real-time analysis across diverse applications in genomics and molecular diagnostics. While natural pores laid the foundation for single-molecule detection, their limited diversity has driven advances in protein engineering and, more recently, *de novo* design to create customizable nanopore sensors. Computational approaches now allow for the design of nanopores with tailored geometries, enhanced stability, and specific molecular recognition functions. Together, these advances are ushering in a new era of programmable nanopore sensors with broad applications in diagnostics and molecular biotechnology.

## 1. Introduction

Nanopore technology is a single-molecule sensing platform that identifies an analyte by detecting the characteristic changes in ionic current as it passes through a nanopore [1]. In this setup, an insulating membrane separates an electrolyte solution into two chambers, with an electrode placed in each. When a voltage is applied across the membrane, no current is detected because the phospholipid bilayer is non-conductive and blocks the flow of ions. However, when a protein nanopore is embedded within this membrane, it creates a channel that allows ions to pass through under the influence of the voltage, generating a constant ionic current [2,3]. Taking DNA sequencing platform as an example, if single-stranded DNA is introduced into the solution on the side of the negative electrode, its inherent negative charge will cause it to be drawn through the nanopore, whose diameter is typically only a few nanometers and is dimensionally matched to the scale of a DNA strand. As the DNA translocases sequentially through the pore, the different nucleotide bases along the strand obstruct the flow of ions to varying degrees. This causes characteristic fluctuations in the ionic current. These electrical signatures directly reflect the sequence information of the DNA strand. By decoding these characteristic changes in the current, the sequence can be inferred. Therefore, by detecting these current fluctuations, the sequence of the DNA strand can be determined, enabling sequencing in real time [4,5,6].

The key to this technology is the nanopore protein itself, which acts as the core sensing element [7]. The nanopore is a nano-scale passage that acts as a gateway through insulating membrane, they are classified into two main types based on their material, biological nanopores and solid-state nanopores [8,9], the biological nanopores utilize pore-forming proteins as channels embedded within a lipid layer or an artificial membrane.

Initially, the field relied on a handful of native pore-forming proteins, which were repurposed as single-molecule sensors. These foundational pores proved the viability of technology and laid the groundwork for applications ranging from DNA sequencing to protein and polypeptides detection [10,11,12,13,14,15]. However, progress was inherently constrained by the limited repertoire of proteins found in nature, which often possess geometries and biophysical properties that are suboptimal for specific analytical tasks. This spurred a wave of creative protein engineering, where researchers learned to “renovate” existing structures —modifying pore dimensions, tuning surface chemistry, and fusing functional domains to enhance performance and create novel functions [11,16,17,18,19,20,21,22,23]. The journey of biological nanopores has been one of remarkable progress, evolving from the initial discovery and characterization of natural protein channel to the sophisticated engineering of complex molecular machines [24,25].

Now the functional protein, moving the field beyond discovery and engineering towards true *de novo* computational design [26,27,28]. By leveraging first-principles design and deep-learning tools [29,30,31,32,33,34,35], scientists now can create entirely novel pore architectures, both β-barrel and α-helical with precisely tailor geometries, stabilities, and functionalities not found in nature [36,37,38,39]. This capability is not only expanding the toolkit of direct-sensing nanopore but is also revolutionizing indirect sensing through the creation of bespoke binders for small molecules and proteins [40], which can be seamlessly integrated into nanopore platforms. We explore how these powerful design capabilities are being harnessed to create a new generation of programable nanopore sensors. Together, these advances are paving the way for bespoke molecular tools with unprecedented potential in diagnostics, proteomics, and fundamental biophysics.

In this review, we provide a comprehensive comparison of native, engineered, and *de novo* nanopores (Table 1). We begin by examining native nanopores, detailing their intrinsic structural characteristics, performance, and established applications. Following this, we discuss engineered nanopores, investigating how rational design applied to natural scaffolds enhance their capabilities, leading to customized nanopores with superior single-molecule resolution and novel functionalities for both DNA and protein analysis. Finally, we focus on the field of *de novo* designed nanopores, where we delve into various design methodologies and their corresponding achievements. Through this structured approach, we aim to provide a clear understanding of the evolution, current state, and future directions in nanopore development.

## 2. Native Nanopores

Scientists have identified and engineered a diverse repertoire of naturally occurring pore-forming proteins that can function as highly sensitive, single-molecule sensors [41]. This toolkit of natural pores is broadly categorized into two major structural classes—β-barrels and α-helical bundles—each with distinct architectures that dictate their optimal applications.

Indeed, the nanopore landscape has been largely dominated by β-barrel proteins. Their prevalence is directly attributable to their field-defining performance in DNA and RNA sequencing. The key to their success lies in their geometry: a rigid, well-defined lumen that narrows to a critically short and narrow constriction zone. In contrast, transmembrane pores formed from α-helical bundles represent a distinct architectural paradigm and are more commonly employed for the detection of intact proteins [42].

This fundamental divergence highlights that different nanopore proteins possess profoundly different characteristics [43]. The choice of a nanopore is not arbitrary but is a strategic decision based on the intended analyte and sensing goal. The structural distinctions—from the short, narrow constrictions of β-barrels ideal for sequencing to the large, accommodating vestibules of α-helical pores perfect for protein trapping—are precisely what enable the functional specialization and expanding versatility of the nanopore platform.

### 2.1. Multimeric β-Barrel Pores for High-Resolution Sequencing

**Figure 1:**
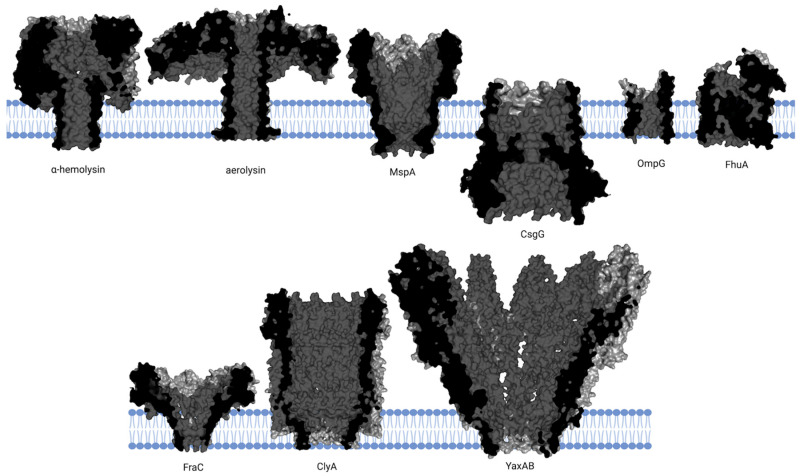
Structures of pore-forming proteins embedded in lipid bilayers. The cross-sectional structures of several pore-forming proteins embedded in lipid bilayers are shown. The proteins include α-hemolysin (PDB: 7AHL), aerolysin (PDB: 5JZT), MspA (PDB: 1UUN), OmpG (PDB: 7Q5C), FhuA (PDB: 1QFG), CsgG (PDB: 4Q79), FraC (PDB: 4TSY), ClyA (PDB: 2WCD), and YaxAB (PDB: 6EL1), each displayed in dark gray with the lipid bilayer highlighted in blue. Created with BioRender.com (accessed on 13 October 2025).

The first biological nanopore protein used for sensing was α-hemolysin, a pore-forming toxin secreted by *Staphylococcus aureus* (Figure 1). It forms a mushroom-shaped homoheptameric complex in lipid bilayers, with an inner pore diameter ranging from 14 to 24 Å [44]. Its narrowest constriction, measuring about 14 Å, is sufficient to accommodate the translocation of single-stranded DNA but sterically excludes double-stranded DNA [45]. However, the protein’s utility for high-resolution sequencing is limited because its β-barrel is too long to distinguish individual nucleotides. The stem of the pore spans approximately 5 nm, a length far exceeding the internucleotide distance of a DNA strand. As ssDNA passes through the nanopore, the resulting ionic current is influenced by a cohort of roughly 12 nucleotide bases simultaneously. This signal averaging effect leads to poor differentiation between adjacent bases, thereby lowering the overall sequencing resolution [46]. Beyond nucleotide detection, engineered mutants of α-hemolysin have been successfully repurposed to function as sensors for other analytes, such as divalent metal ions and organic analytes [47,48,49].

Expanding the toolkit of biological nanopores beyond α-hemolysin, researchers have successfully employed other bacterial toxins such as aerolysin (from *Aeromanas hydrophila*) and cytotoxin K (from *Bacillus cereus*) [50,51,52] (Figure 1). Aerolysin, in particular, presents a compelling alternative, while it shares the mushroom-shaped, heptameric structure of α-hemolysin, its pore possesses a distinct and highly advantageous geometry, a long, narrow lumen with a net charge [53]. When individual amino acids are covalently ligated to a carrier molecule that guides them through the pore, such as negatively charged peptide, the wild-type aerolysin channel can unambiguously resolve all twenty standard proteinogenic amino acids [54]. The unique electrical current signature generated by each amino acid as it transiently interacts with the pore’s charged lumen enables its identification.

MspA is a nanopore protein identified in *Mycobacterium smegmatis*, known for its funnel-shaped structure that offers significant advantages over α-hemolysin. Its constriction region has diameter of 1.3 nm and is only about 1 nm in thickness (Figure 1), so MspA is uniquely suited for high-resolution sensing applications [55]. However, the wild-type MspA is not capable of detecting the ionic current signals of single-stranded DNA. To address this, researchers have engineered MspA by mutating negatively charged amino acids on the inner surface of the pore to either positively charged or neutral amino acids. This modification allows the nanopore to distinguish the characteristic signals of the four nucleotides. Compared to α-hemolysin, the engineered MspA generates larger current differences for different nucleotides [56].

Another cornerstone of modern nanopore technology is CsgG, a nonameric peptide transporter involved in the bacterial curli secretion system (Figure 1). CsgG serves as the foundation for Oxfold Nanopore Technologies’ R9 nanopore, updated in 2016, which boasts optimized signal-to-noise ratio and nucleotide resolution [57]. The CsgG protein forms a ring-like structure with a wide, 40 Å external opening that narrows to a constriction of approximately 10 Å in diameter. This critical sensing region is defined by three stacked, concentric rings of amino acid side chains-phenylalanine, asparagine, and tyrosine-which interact directly with the translocation analyte [58].

### 2.2. Monomeric β-Barrel Pores for Versatile Protein Detection

While β-barrel nanopores like MspA and CsgG, often featuring large soluble domains, are primarily used for DNA sequencing, simpler monomeric β-barrel pores like OmpG and FhuA have been adapted for different sensing paradigms, offer unique advantages for sensing applications [59,60] (Figure 1). OmpG forms a relatively cylindrical pore. Its structure includes flexible loops extending from the cis entrance that can act as molecular gates; this property has been exploited to detect binding events, such as the recognition of streptavidin, by monitoring characteristic current blockades [61]. FhuA, a larger β-barrel, has been engineered as a highly versatile and generalizable platform for protein detection. The key innovation involves covalently attaching an external protein binder to the FhuA nanopore. This design uses an antibody-mimetic protein called a monobody tethered to the pore’s entrance. This modular design allows the sensor to be adapted for countless different protein targets simply by swapping the monobody binder, without altering the core nanopore architecture [62].

### 2.3. α-Helical Pores for Large Protein Trapping

In contrast to the β-barrel family, α-helical nanopores offer distinct structural features, often forming large, stable channels with expansive vestibules ideal for trapping and analyzing entire proteins. Examples include Cytolysin A (ClyA), Fragaceatoxin C (FraC), and YaxAB (Figure 1).

ClyA assembles as a dodecamer with a height of about 130 Å. The pore constricts to a diameter of 3.3 nm at its narrowest point. Its most notable feature is a large vestibule that can trap proteins in the 20–50 kD size range, leading to long-lived blockade events that are characteristic of the trapped protein’s identity [63,64].

FraC is another α-helical pore from the actinoporin family that self-assembles into a symmetrical, funnel-shaped-shaped octameric particle. The pore measures approximately 110 Å in outer diameter and 70 Å in height, its inner diameter varies significantly, from 60 Å at the upper (cis) vestibule down to its narrowest point of about 16 Å at the trans constriction. The large cis opening, about 6 nm in diameter, facilitates analyte entry and guides them towards a sharp, narrow constriction of only 1.6 nm near the trans side. Notably, the pore lumen exhibits a negative electrostatic potential, making FraC a highly cation-selective channel [65,66].

YaxAB: the YaxAB toxin complex shares similarities with FraC, forming a large, conical-shaped pore with a 15 nm cis-opening and a 3.5 nm trans-constriction. This geometry is exceptionally well-suited for accommodating and characterizing a wide range of protein sizes [67,68]. Crucially, the conical shape dictates the depth of protein penetration. Smaller proteins can enter deeper into the constriction region, resulting in a larger fractional current blockade compared to larger proteins that are sterically restricted to the wider vestibule. The current blockade is inversely proportional to the size of the trapped protein.

### 2.4. Metagenomic Mining

The future of nanopore technology lies in moving beyond the known portfolio of proteins. Computational methods are accelerating the discovery of entirely novel nanopore proteins [69,70]. By leveraging artificial intelligence and deep metagenomic mining, researchers are now identifying entirely new families of pore-forming proteins [71]. A recent study used AlphaFold to screen deep-sea metagenomic databases, identifying several novel protein families with unique sequences and structures. These candidates exhibit less than 50% sequence homology to any known pore proteins, promising new capabilities for sensing and sequencing [72]. Structural predictions with AlphaFold and subsequent experimental validation confirmed that these proteins can self-assemble into homomultimeric nanoscale channels, dramatically expanding the structural and functional diversity of the nanopore toolkit.

## 3. Engineered Nanopores

While the discovery of novel native nanopores continues, the available pool remains limited. These natural pores, though robust, often possess fixed pore geometries, charge distributions, and chemical functionalities. This inherent lack of precise control over their sensing characteristics often limits their performance in demanding single-molecule analyses. This has driven a significant effort in protein engineering to create customized nanopores with superior single-molecule resolution and novel functionalities for both DNA and protein analysis.

For DNA sequencing, a key conceptual advance was the “two heads are better than one” principle proposed by Hagan Bayley and colleagues. This design involves creating a nanopore with distinct recognition sites (R1 and R2) [73]. As a nucleic acid strand translocates, the two sites can interact with the four nucleobases to generate up to 16 distinct current states, providing far more information than the four signals from a single-site pore. Furthermore, this allows each base to be read twice, once at R1 and again at R2, creating an internal cross-checking mechanism that enhances the overall quality and accuracy of the sequence data.

A prime example of this principle in action was demonstrated by the Oxford Nanopore Technologies team in 2020. They engineered a composite nanopore by combining the CsgG pore with the N-terminal 35 amino acids of its chaperone, CsgF, creating a stable dual-constriction channel [74]. The N-terminus of CsgF penetrates deep into the CsgG β-barrel, where its first four residues (G1 to T4) form a complex hydrogen-bond network with residues Q153, D155, T207, and N209 of the CsgG lumen, a strictly conserved NPXFGG motif allows the CsgF N-terminus to project into the CsgG barrel, creating a primary channel constriction 15 Å wide, with residue N17 at its apex. From this point, the CsgF peptide returns toward the barrel’s rim via a 13-residue helix, forming a highly stable, non-covalent complex that can withstand temperatures up to 70 °C. This exceptional thermal stability translates directly to more robust and reliable sequencing devices, and the dual-constriction architectures significantly improves the resolution of oligonucleotides [75,76].

Applying nanopores to protein sequencing presents substantially greater challenges than DNA sequencing. Unlike uniformly negatively charged nucleic acids, amino acids exhibit diverse charges, varying hydrophobicity and side-chain structures [77]. Therefore, nanopores for protein analysis must be engineered not only with an optimized constriction region but also with consideration for the pore’s overall length and its ability to process and transport a polypeptide chain [78,79].

A groundbreaking approach to tackle this was demonstrated in the bottom-up fabrication of a proteasome-nanopore [80]. This molecular machine was built by first creating a stable, low-noise β-barrel sensor. The disordered region of a mammalian proteasome activator (REGα, or 11S activator) was replaced with the β-barrel transmembrane domain from the anthrax protective antigen nanopore, chosen for its high thermodynamic stability and tolerance to sequence substitutions. This engineered REG-nanopore was then fused to the 20S proteasome from *Thermoplasma acidophilum*, which is composed of four stacked rings of α- and β-subunits. The resulting proteasome-nanopore complex enables two distinct modes of protein analysis: (1) Thread and read: the proteasome unfolds a target protein and threads the linearized polypeptide through the nanopore for analysis. (2) Chop and drop: the proteasome unfolds and degrades the protein, releasing the resulting peptides into the nanopore for detection. The strategy of coupling a nanopore to a soluble, ring-like oligomeric protein complex provides a new paradigm for designing molecular machine with advanced functions [81].

**Figure 2:**
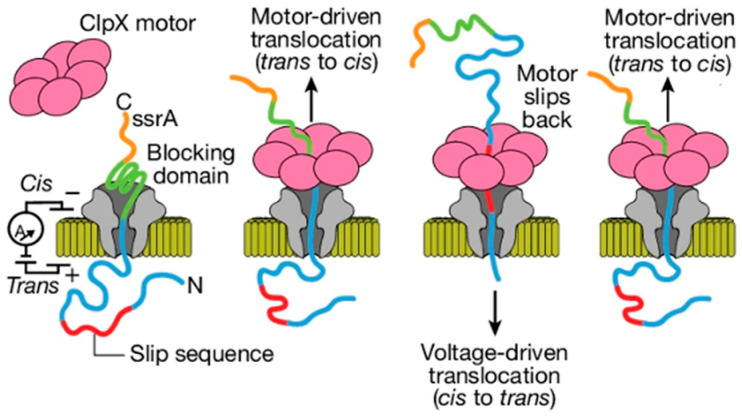
Engineering strategies of natural nanopores. ClpX-assisted thread-and-read strategy: proteins are first electrophoretically transferred through CsgG to the trans side, then pulled back to the cis side by ClpX. Figure 2 from ref. [25], is used under a CC BY 4.0 license (http://creativecommons.org/licenses/by/4.0/ (accessed on 13 October 2025)).

A separate groundbreaking approach to threading and reading was demonstrated by coupling the CsgG nanopore with the protein unfoldase ClpX (Figure 2) [25]. This molecular motor was shown to actively pull long protein strands through the nanopore in a controlled, multi-pass fashion, offering another powerful paradigm for single-molecule protein analysis.

## 4. *De Novo* Design of Nanopores

As our understanding of single-molecule biophysics deepens and the field of protein design rapidly advances, the potential to design nanopores with customized architectures and sensing capabilities has surged [82,83]. Traditionally, nanopore sensing is categorized into two modes: direct sensing, where the analytes itself produces a signal as it translocates, and indirect sensing, where the binding of an analyte to a receptor tethered to the pore modulates the ionic current [84]. Across both sensing paradigms, computational design is increasingly shaping the next generation of nanopore technologies. The increasing success rate in designing specific binders for both large target proteins and small molecules now allows for the bespoke engineering of nanopores tailored to virtually any analyte [85]. This marks a true paradigm shift—from refining nature’s pores to designing entirely new ones from scratch, opening access to structures and functions nature never evolved [86]. Although the practical application of these designed pores in high-throughput sequencing is still on the horizon, many have already demonstrated robust single-molecule detection capabilities.

### 4.1. De Novo Design of Transmembrane β-Barrel Nanopores

Comparing to the native nanopores, the structure created through *de novo* design are, for now, relatively simple [87]. Nonetheless, they represent a monumental leap forward in custom sensor engineering. The design of transmembrane β-barrels, in particular, has seen remarkable progress in the last five years (Table 2).

**Figure 3:**
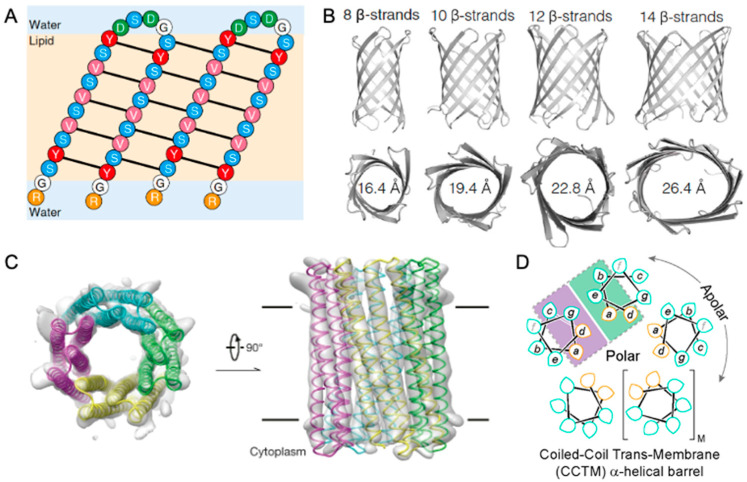
*De novo* designed nanopore proteins. (**A**) Amino acid sequence of the designed SV28 nanopore, with black lines indicating backbone hydrogen bonding. (**B**) Transmembrane β-barrels of varying strand numbers and pore diameters designed using the blueprint scheme. (**C**) Comparison of the cryo-EM density map (gray surface) and structural model (colored ribbon) of the TMH4C4 protein, showing a pore of ~10 Å diameter. (**D**) Heptad repeat schematic with side-chain orientations (teardrop tips); hydrophobic (cyan) and polar (orange) residues are highlighted, with designed interfaces for bZIP scoring marked in purple and green. (**A**) from ref. [88], (**B**) from ref. [90], is used under a CC BY 4.0 license (http://creativecommons.org/licenses/by/4.0/ (accessed on 13 October 2025)). (**D**) reprinted with permission from ref. [94], American Chemical Society.

The design of the β-hairpin peptide SV28 provided a generalized framework for creating transmembrane barrels (Figure 3A) [88]. This strategy was based on a modular design strategy and precise control over electrostatic interactions. The peptide was partitioned into functional units: β-strands, which form the core transmembrane regions designed to be 10 amino acids long to match the thickness of a lipid bilayer; β-turn, a connecting motif between the two β-strands where a negative charge was introduced, while positive charges were placed at the N- and C-termini to electrostatically guide membrane insertion and orientation; and stabilizing elements, including aromatic residues and a hydrogen-bond network critical for stabilizing the final transmembrane structure. The initially designed SV28 peptides self-assembled into a heterogenous population of pores with diameters ranging from 1.7 to 6.3 nm. However, through the rational introduction of a glycine “kink” mutation, the assembly was restricted to a single, monodisperse 1.7 nm pore, demonstrating precise control over the final structure [95].

The Rosetta-based “blueprint” method has enabled the design of β-barrel proteins with customized sizes and shapes [96]. This approach defines the barrel’s geometry using key parameters: the number of strands, the shear number (the stagger between adjacent strands), and the barrel length. The process begins with a 2D blueprint that explicitly defines the arrangement of β-strands, the hydrogen-bonding network, and the positions of structural motifs like β-bulges and glycine kinks. Rosetta then converts this 2D plan into a 3D backbone, which is subsequently optimized using relaxation protocols [89,97].

A critical innovation in this method lies in sequence design. While the pore lumen is lined with hydrophilic residues, the membrane-embedded region is not composed exclusively of hydrophobic amino acids. Instead, a degree of energetic “frustration” is deliberately introduced by including some less favorable residues. This prevents the pores from forming irreversible aggregates within the membrane, a common failure mode. The use of the deep-learning tool ProteinMPNN [34], which has implicitly learned the complex sequence-structure relationships of transmembrane proteins, has further improved the success rate compared to traditional Rosetta sequence design [98]. This blueprint method has successfully produced a diverse array of pores, including 8- to 14-stranded barrels with triangular, oval, and rectangular cross-sections, whose electrical conductance is comparable to that of natural pores (Figure 3B) [90]. This ability to sculpt pore shapes is the first step toward creating binding pockets for the specific detection of diverse small molecules.

A more recent and powerful strategy, the parametric method, overcome the reliance on expert-driven manual blueprints. This approach uses global parameters, such as strand number and shear number, to automatically generate an idealized backbone. This initial structure is then refined using deep-learning methods like RFjoint2 or RFdiffusion to correct any non-ideal geometries. ProteinMPNN is then employed to generate a highly compatible sequence.

This automated pipeline has proven to be remarkably effective, with 10 of 81 designs successfully inserting into lipid membranes—a success rate for exceeding that of the traditional blueprint method. This approach not only recapitulated the design of 12- and 14-stranded pores but also achieved the first-ever *de novo* design of a 16-stranded β-barrel [91].

### 4.2. De Novo Design of α-Helical Nanopores-from Ion Channels to Tunable Pores

The design of transmembrane α-helical proteins has proven to be a remarkably diverse and fruitful area of research, yielding structures ranging from simple ion channels to larger conducting pores, offering unique opportunities for custom-designed single-molecule sensing (Table 2). These achievements are critical steps toward the ultimate goal of creating fully “rationally designed nanopores” with bespoke functionalities.

Early forays into α-helical membrane protein design focused on relatively compact structures, such as single-pass helices or small oligomeric bundles. Pioneering work in this domain includes the design of a homo-tetrameric four-helix bundle capable of transporting Zn^2+^ ions across the membrane [99]. In this design, two helices form a table interacting domain, which then dimerizes to create the functional channel.

A cornerstone of this progress is the partitioned design strategy implemented in computational tools like Rosetta [100]. This approach explicitly accounts for the distinct chemical environments a membrane protein inhabits by dividing it into three zones: the aqueous phases (cytoplasmic and extracellular), the hydrophobic lipid core, and the protein’s own internal core. Using a specialized energy function calibrated for the unique biophysics of the membrane, this algorithm searches for the lowest-energy amino acid sequence that satisfies the chemical requirements of all three regions. Alongside advances in designing polar interactions within protein cores [101], this strategy has enabled accurate control over protein topology and transmembrane orientation, facilitating the construction of more complex membrane protein architectures [92].

Moving beyond compact channels to larger, conductive pores presented significant challenges. Pores require larger solvent-accessible lumens, implying lower density of intramolecular interactions and greater surface area exposed to the membrane-inherently less stable than tightly packed helical bundles. The parametric design method has emerged as a powerful tool to meet this challenge by enabling precise control over the arrangement of α-helices. By systematically tuning the geometric parameters of the Crick equation [102], researchers can generate idealized backbones with exact control over pore size and shape.

This method has been used to generate complex architectures, such as a 12-helix C6-symmetric hexamer and a 16-helix C8-symmetric octamer, both composed of two concentric rings of α-helices. The sequence design for these barrels often employs a sophisticated two-step strategy. First, a stable, water-soluble version of the protein is designed, using extensive hydrogen-bond networks to reinforce the interactions between helices. Once this water-soluble structure is experimentally validated, its surface residues are computationally replaced with hydrophobic amino acids to drive membrane insertion. This approach has successfully yielded functional pores like TMHC6, which exhibits ion selectivity, and TMH4C4, which is permeable to small molecules (Figure 3C) [93].

Beyond energy function-based methods, an alternative and elegant strategy relies on a set of simplified chemical rules to design functional α-helical barrels. This approach leverages the heptad repeat (abcdefg), a structural hallmark of α-helices (Figure 3D) [103]. By designing short peptides with hydrophobic residues at the helical interface positions and hydrophilic residues elsewhere, researchers have created water-soluble peptides driven by the burial of their hydrophobic faces. By using the bZip scoring function, complementary g, a, d, e interfaces are selected, enabling the self-assembly of stable pentameric, hexameric, and heptameric barrels. The specific residues at the g, a, d and e positions have been shown to be critical in determining the final oligomeric state and specificity of assembly [104,105].

These stable, water-soluble α-helical barrel serves as ideal templates for creating transmembrane ion channels via a process known as modular surface remodeling [94,106]. Starting with a soluble protein of known stability and appropriate size, only the surface is re-engineered. Hydrophobic residues like tryptophan and leucine are introduced onto the exterior surface to facilitate membrane insertion, while the N- and C-termini are adjusted to promote correct orientation. Critically, specific polar residues can be rationally placed within the channel lumen to confer ion selectivity. This modular approach significantly increases the success rate of designing functional membrane proteins by building upon a pre-validated, stable scaffold.

The design of transmembrane α-helical barrels has now progressed from constructing basic structures to engineering dynamic, functional systems. For instance, by altering amino acids at specific positions, the oligomeric state of self-assembling barrels can be precisely controlled. Furthermore, transmembrane peptides based on the coiled-coil heptad repeat have been shown to form conductive channels that exhibit complex, voltage-dependent behavior. These artificial channels often display two distinct conductance states: a low-conductance state, which corresponds to the computationally designed structure, and a high-conductance state. The fact that high-conductance state is similar across different peptide designs suggests that the channels are not rigid but can dynamically and discretely expand in size. This discovery of conformational plasticity reveals that *de novo* designed pores can be much more than static conduits, opening the door to engineering channels with sophisticated gating and regulatory functions [94].

### 4.3. De Novo Designed Target Binding Proteins for Indirect Nanopore Sensing

The remarkable progress in *de novo* computational protein design, particularly in creating high-affinity binders for diverse targets-encompassing both small molecules and large proteins [107,108,109,110,111], represents a transformative leap for nanopore-based indirect sensing technologies, where the nanopore acts as a transducer to convert a specific binding event at its orifice into a clear electrical signal. The capability directly addresses a core challenge: translating molecular recognition events into robust, quantifiable electrical signals within a nanopore platform.

Indirect protein sensing has traditionally relied on coupling natural receptors to nanopores and detecting changes in ionic current upon target binding [62,112,113]. Computational protein design now enables the creation of synthetic binders with programmable specificity and tunable properties. In principle, a designed binder for any target could be incorporated into a nanopore sensor, freeing researchers from dependence on naturally evolved or immunization-derived receptors. High-affinity binders have already been successfully designed against highly challenging and clinically relevant targets, such as the neurotoxin and the SARS-CoV-2 receptor-binding domain (RBD), TNF-α, with affinities reaching the picomolar-to-nanomolar range [40,114,115,116]. These designed binders are comparable in size to nanobodies and exhibit exceptional thermal stability, making them ideal components for robust, field-deployable sensor platforms.

Integrating computationally designed binders with nanopores could offer an effective strategy for protein detection. These binders can be precisely engineered in size and orientation, allowing fine control over their spatial arrangement at the pore entrance. Their compact structure minimizes interference with ionic flow, maintaining a stable baseline current crucial for sensitive signal detection. When tethered externally, the binding interface can be readily adjusted to remain solvent-exposed and optimally oriented for efficient capture of target proteins from solution.

Similarly, incorporating *de novo* designed binders for small molecules represents a promising strategy for molecular detection. Designing such binders is inherently challenging: unlike large proteins, small molecules present limited surface area and few distinctive chemical features, constraining the ways a protein can engage and recognize them. Overcoming these constraints is key to expanding the scope of programmable nanopore sensors for small molecule detection.

In recent years, significant progress has been made in *de novo* designing proteins that bind specific small molecules. A notable computational approach is the “van der Mer” (vdM) method [117], which provides a powerful framework for identifying favorable residue–ligand interactions. Conceptually analogous to libraries of protein side-chain rotamers, the vdM methodology extends this principle significantly. Its core innovation lies in directly linking the backbone conformation of each amino acid residue to the statistically preferred spatial positions of interacting chemical functional groups. Large datasets of high-resolution protein crystal structures are systematically mined. Within these structures, amino acid residues engaging specific chemical moieties (e.g., hydroxyls, carbonyls, aromatic rings) are identified. The geometric coordinates of both the residue’s backbone atoms and the interacting ligand functional group are then extracted and subjected to rigorous clustering analysis [118]. The efficacy of this approach was robustly demonstrated in the design of a high-affinity protein binder for the drug Rucaparib [119].

In the era of deep learning, a diverse suite of tools now empowers ligand-binding protein design—from backbone generation to sequence calculation and in silico selection—greatly improving both success rates and design efficiency. Highly integrated computational pipelines have been used to design binders for structurally diverse small molecules [120,121]. In this study, the authors employed “pseudocycles” as the foundational scaffolds, a class of cyclic protein architectures comprising repeating structural motifs pseudo-symmetrically arranged around a central cavity or pore. The computational pipeline for this approach is highly sophisticated: AlphaFold and ProteinMPNN are used to generate novel pseudocycle scaffolds based on the desired pocket geometry, while Rosetta and the specialized tool LigandMPNN are used to optimize the sequences specifically for high-affinity binding to the target small molecules [29,34,122,123]. This approach has successfully generated high-affinity binders for diverse ligands, including challenging polar and flexible targets like methothrexate and the compound thyroxine. The ability to design binders *de novo* for such varied targets underscores the power and generality of this methodology [124].

**Figure 4:**
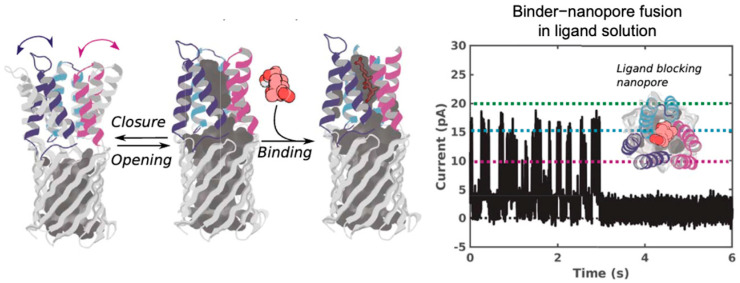
Indirect sensing of proteins and small molecules using nanopores. Ligand-sensing *de novo* nanopore incorporating a cholic acid binder (CHD_r1). Three helical hairpins of the binder were inserted into different loops of the designed β-barrel. In the absence of ligand, transient interactions lead to current fluctuations, whereas ligand binding stabilizes the interaction and produces prolonged current blockades. Different current levels (0, 10, 15, and 20 pA) are denoted by black, magenta, teal, and green dashed lines, respectively. Figure 4 from ref. [124] is used under a CC BY 4.0 license (http://creativecommons.org/licenses/by/4.0/ (accessed on 13 October 2025)).

The ligand binding capability has been directly translated into a functional nanopore sensor. Researchers successfully integrated a *de novo*-designed cholic acid binder, composed of three helical hairpins, into three distinct locations within a TMB12_3 β barrel nanopore (Figure 4) [124]. The resulting chimeric sensor demonstrated that the presence of cholic acid causes the binder to engage its target and physically block the nanopore channel. This effectively converted the small-molecule binding event into a discrete, all-or-none electrical signal, enabling the highly sensitive and specific detection of cholic acid.

The field of small molecule-binding protein design is advancing rapidly thanks to new AI tools like AlphaFold3 and all-atom diffusion models. These technologies are making protein design faster and more effective. AlphaFold3 precisely predicts how proteins and small molecules bind [125], All-atom diffusion models can directly generate new proteins tailored to a specific molecule, a significant leap beyond traditional methods [120]. Together, these advances could enable the creation of highly selective, *de novo*-designed receptors for nanopore sensors, dramatically expanding their capability for small-molecule detection.

However, the practical realization of *de novo* nanopores remains challenging. Designing membrane proteins themselves, particularly large pore-forming proteins suitable for nanopore sensing, presents a major hurdle, with successful cases being notably scarce. Controlling the magnitude of the ionic current and mitigating signal noise are crucial for high-performance sensing, and there is a critical difficulty lies in achieving stable and controllable membrane insertion and orientation within lipid bilayers. These functions are often hard to predict accurately using computational methods alone. In addition, *de novo* designed nanopores often lack the structural complexity and topological features characteristic of natural pores, limiting their functional versatility. Finally, translating complex in silico designs into reproducible experimental results remains a formidable challenge, often complicated by discrepancies between idealized theoretical models and the realities of synthesis, folding, and characterization within a biological environment. Addressing these multifaceted challenges will be essential for bridging computational innovation with practical single-molecule sensing applications.

## 5. Conclusions

In summary, the field of biological nanopore technology has rapidly transformed from a foundational single-molecule sensing platform, reliant on a handful of natural protein channels, into a highly diverse, engineered, and computationally driven domain. While natural nanopore proteins served as the indispensable cornerstones of this technology, the subsequent engineering of these proteins markedly improved the capabilities of natural nanopores and broadened their applications. Pushing beyond the limits of natural and engineered systems, computationally driven *de novo* design marks a revolutionary paradigm shift. Computational approaches have led to the creation of a diverse arsenal of artificial channel proteins, non-existent in nature, capable of selective ion transport and shape-based molecular recognition. A particularly powerful application of this new paradigm is the development of indirect sensing platforms. By integrating computationally designed binder proteins—which can be engineered to capture specific small molecules or target proteins—into the nanopore lumen, researchers can now monitor binding events that are otherwise invisible to direct detection, a significant advantage for diagnostics and proteomics. The integration of computational protein design with nanopore technology is driving the field toward its next frontier—the development of fully programmable nanopore sensors, in which both structure and function can be rationally engineered to meet virtually any molecular sensing challenge.

## Figures and Tables

**Table 1:** Comparison and characterization of native, engineered, and *de novo* designed nanopores in terms of structure, performance, application scope, advantages, and limitations.

Category	Structure	Performance	Application Scope	Key Advantages	Limitations
Native Nanopores	Mushroom- or funnel-shaped pores with varying diameters	Stable and reproducible single-molecule signals	DNA/RNA sequencing; protein detection	High signal stability; biologically robust; wide range of sizes	Limited natural pool; restricted tunability
Engineered Nanopores	Multi-domain fusion based on native scaffolds	Enhanced resolution and gating dynamics; superior single-molecule performance	Advanced DNA and protein analysis and sequencing	Achieves customized functional enhancement while maintaining native pore stability	Constrained by natural backbone; limited modification space
*De novo* designed Nanopores	Cylindrical pores with tunable geometry and pore diameter; incorporation of novel sensing components	Full control over architecture, stability, and binding properties	Specific molecular recognition and custom biosensing	Breaks limitations of native and engineered pores; enables novel functionalities	Difficult to achieve robust, stable, and reproducible pore function; design and validation remain complex

**Table 2:** Overview of representative strategies for *de novo* design of nanopores. Summary of representative *de novo* nanopore design strategies and their outcomes, highlighting key computational approaches and breakthroughs in achieving stable and functional synthetic nanopores. Data summarized from refs. [88,89,90,91,92,93,94].

	Design Method	Key Parameters/Strategy	Outcomes	References
β-barrel nanopores	Rational design	β-strands; β-turn; stabilizing elements; hydrogen-bond network.	SV28 nanopore; SVG28 nanopore.	[88]
	Rosetta design	Parameters: number of strands (n); shear number (s); barrel length (I) 2D blueprint → 3D backbone → sequence design.	8–14-stranded barrels; triangular, oval, and rectangular cross-sections, electrical conductance comparable to natural pores.	[89,90]
	Parametric design & Deep learning	Parameters: strand number, shear number; strand length refined with RFjoint2 and RFdiffusion	Higher success rate; 16-stranded pore.	[91]
α-helical nanopores	Parametric and Rosetta	3 regions: aqueous, hydrophobic lipid core, internal core.	Atomic-level control over protein topology and transmembrane orientation.	[92]
	Parametric and Rosetta	Tuning Crick equation parameters for pore size and shape.	TMHC6 (ion selective), TMH4C4 (small molecule permeable).	[93]
	Rational Design	Heptad repeat (abcdefg)	Stable self-assembling barrels	[94]

## Data Availability

No new data were created or analyzed in this study. Data sharing is not applicable to this article.
